# Diagnosis and management of 5-fluorouracil (5-FU)-induced acute leukoencephalopathy: lessons learnt from a single-Centre case series

**DOI:** 10.1186/s43046-022-00117-4

**Published:** 2022-05-23

**Authors:** Neethu Jose, Anjana Joel, Rajesh Joseph Selvakumar, Jebakarunya Ramireddy, Ajoy Oommen John, Josh Thomas Georgy, Ashish Singh, Thomas Samuel Ram

**Affiliations:** 1 Department of Radiotherapy (Unit 1), CMC Vellore, Vellore, 632004 India; 2 Department of Medical Oncology, CMC Vellore, Vellore, 632004 India; 3 Department of General Surgery, CMC Vellore, Vellore, 632004 India

**Keywords:** 5-fluorouracil, 5-FU, Fluoropyrimidines, Toxic leukoencephalopathy, Magnetic resonance imaging (MRI)

## Abstract

**Background:**

The administration of 5-fluorouracil (5FU) in the treatment of gastrointestinal (GI) malignancies is associated with common side effects such as mucositis, diarrhoea, and myelosuppression, which are easily managed with supportive measures and dose adjustments. Cardiotoxicity and neurotoxicity are rare but reversible side effects of 5-FU and are treated with withdrawal of the drug and conservative measures. The presenting symptoms of 5-FU-induced leukoencephalopathy are often confusing and pose a diagnostic dilemma in routine clinical practice.

**Methods:**

We report a series of five patients with GI malignancies who developed 5-FU-induced leukoencephalopathy.

**Results:**

All (*n* = 5) had Naranjo scores of 6–7, predictive of 5-FU-related adverse effects, with clinical and radiological findings suggestive of 5-FU-induced encephalopathy as described in prior literature. The median time to onset of symptoms from initiation of 5FU was 3 days (range: 2–4 days). All patients improved after conservative management with complete neurological recovery.

**Conclusion:**

Prompt recognition of this rare yet severe adverse effect of 5-FU-based chemotherapy aids early withdrawal of the offending agent (5-FU) and timely initiation of supportive measures and helps plan alternative oncological interventions.

## Background

**Fig. 1:**
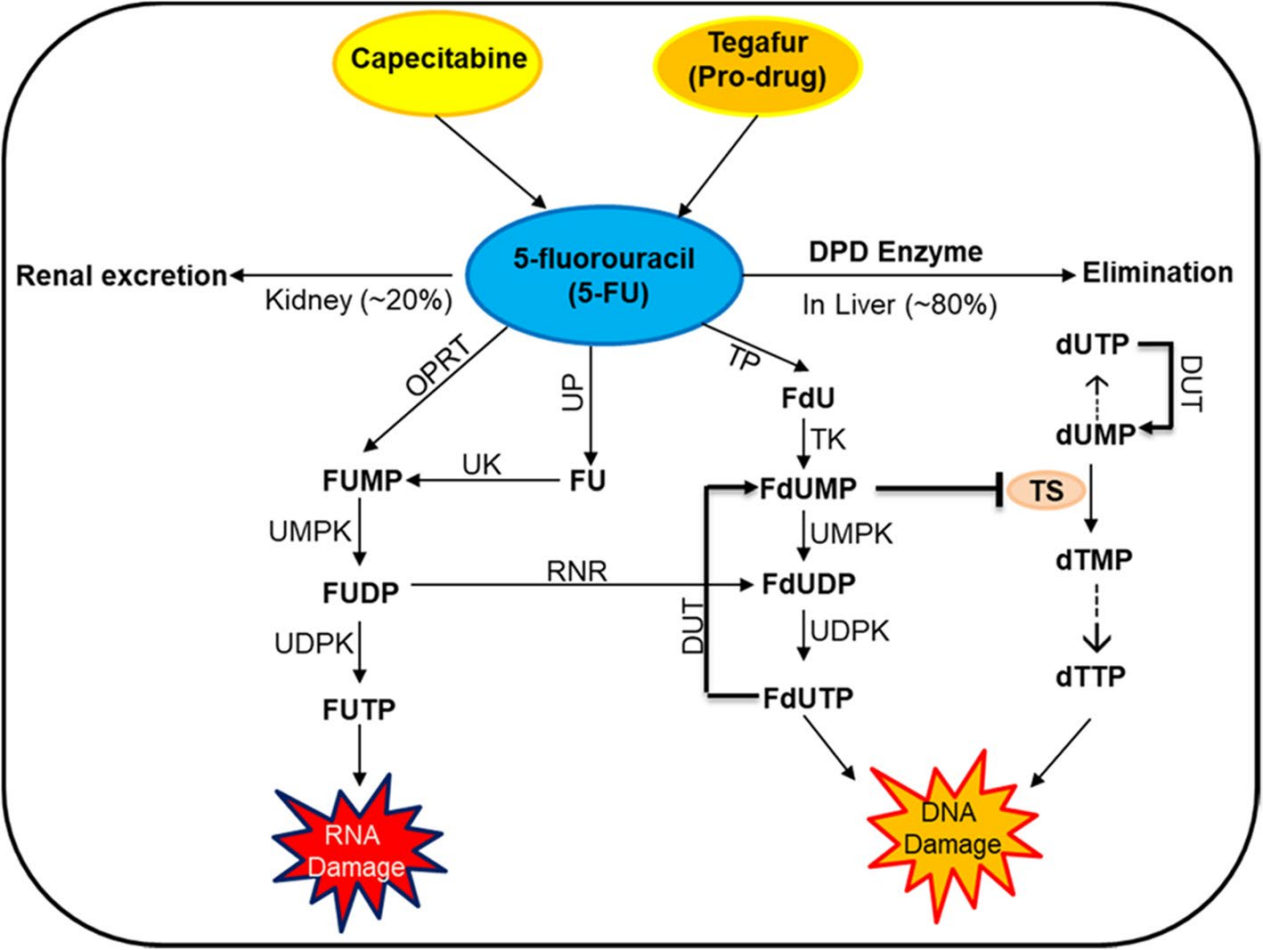
Fluoropyrimidine-based drug metabolic pathway (9)

5-Fluorouracil (5-FU) is a pyrimidine uracil analogue which is the backbone of most chemotherapeutic regimens used in the treatment of gastrointestinal (GI) malignancies; including those of the colon, rectum, stomach, and pancreas [1]. The active metabolite of 5FU, fluorodeoxyuridine monophosphate (Fd-UMP), binds with thymidylate synthetase enzyme through competitive inhibition with uracil and the co-factor resulting in decreased DNA synthesis, repair, and cell proliferation [2] (Fig. 1). Frequently reported side effects of 5-FU including nausea, vomiting, diarrhoea, mucositis, myelosuppression, and cardiotoxicity are attributed to this metabolite [1, 3].

Individuals with an inherited deficiency in DPD (dihydropyrimidine dehydrogenase) enzyme activity are more prone to severe and life-threatening 5-FU-related toxicity in the form of mucositis, neutropenia, and diarrhoea [4, 5]. Neurotoxicity associated with 5-FU is rare and not associated with DPD enzyme deficiency [6–8]. This entity is confirmed by its typical clinical presentation, features on magnetic resonance imaging (MRI), and is managed conservatively with temporary discontinuation of the offending chemotherapeutic agent, i.e. 5-FU.

## Methods

Data of adult patients with GI malignancies who received 5-FU-based chemotherapy at our centre, between January 2017 and December 2019, were captured from the patient records. Acute 5-FU-related toxic encephalopathy was diagnosed in five patients, based on the temporal development of encephalopathy during (or) shortly after completion of 5-FU infusion. This study was approved by the Institutional Review Board (IRB) and Ethics Committee (EC) of our centre.

## Results

The baseline characteristics of these five patients are summarised in Table 1, and individual patients are discussed below.

**Table 1:** Patient characteristics, clinical course and details of fluoropyrimidine rechallenge

Case	Age (years)	Gender	Primary	Regimen	Cycle of onset	Day of onset	5-FU dosing	DPD testing	Naranjo score [9]	Fluoropyrimidine rechallenge (if any)	Follow-up (months)
1	22	Female	Rectum	m-FOLFOX6	1	2	Bolus: 400 mg/m^2^ D1 Infusion: 2400 mg/m^2^ over 48 h	Mutation in exon 2, 13 and 18 (heterozygous)	7	Capecitabine	25
2	30	Male	Rectum	5FU/LV	1	4	Bolus: 400 mg/m^2^ D1 Infusion: 2400 mg/m^2^ over 48 h	Wild	6	Capecitabine	23
3	34	Male	Rectum	5FU/LV	3	3	Bolus: 400 mg/m^2^ D1 Infusion: 2400 mg/m^2^ over 48 h	Wild	6	None	8
4	45	Female	Stomach	FLOT	1	2	Infusion:2600 mg/m^2^ over 24 h	Not done	6	Capecitabine	12
5	60	Male	Rectum	FOLFIRINOX	2	3	Bolus: not given Infusion: 2400 mg/m^2^ over 48 h	Not done	6	Capecitabine	4

### Case 1

**Fig. 2:**
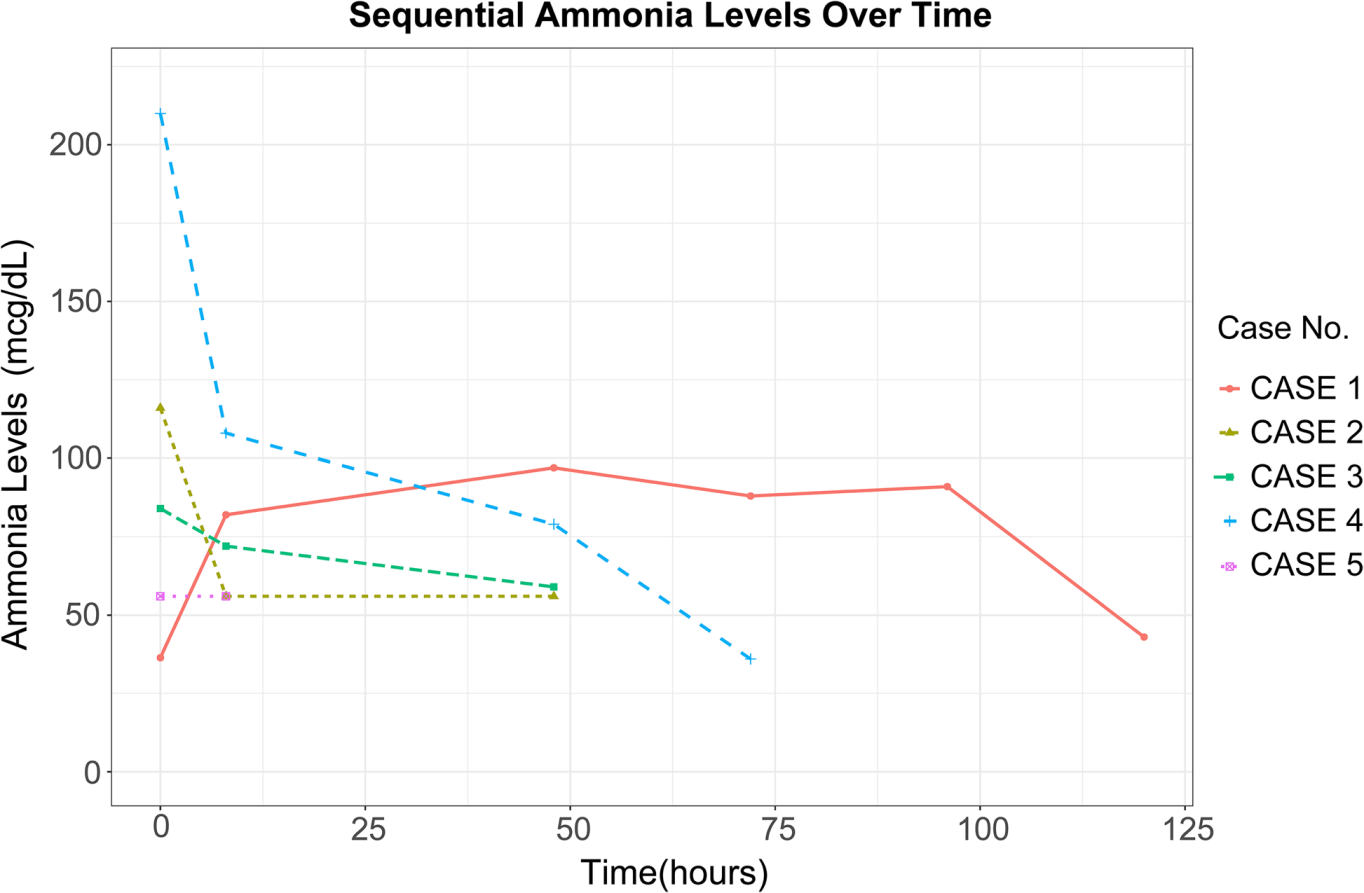
Spider plot of serial serum ammonia levels for all patients (*n* = 5)

**Fig. 3:**
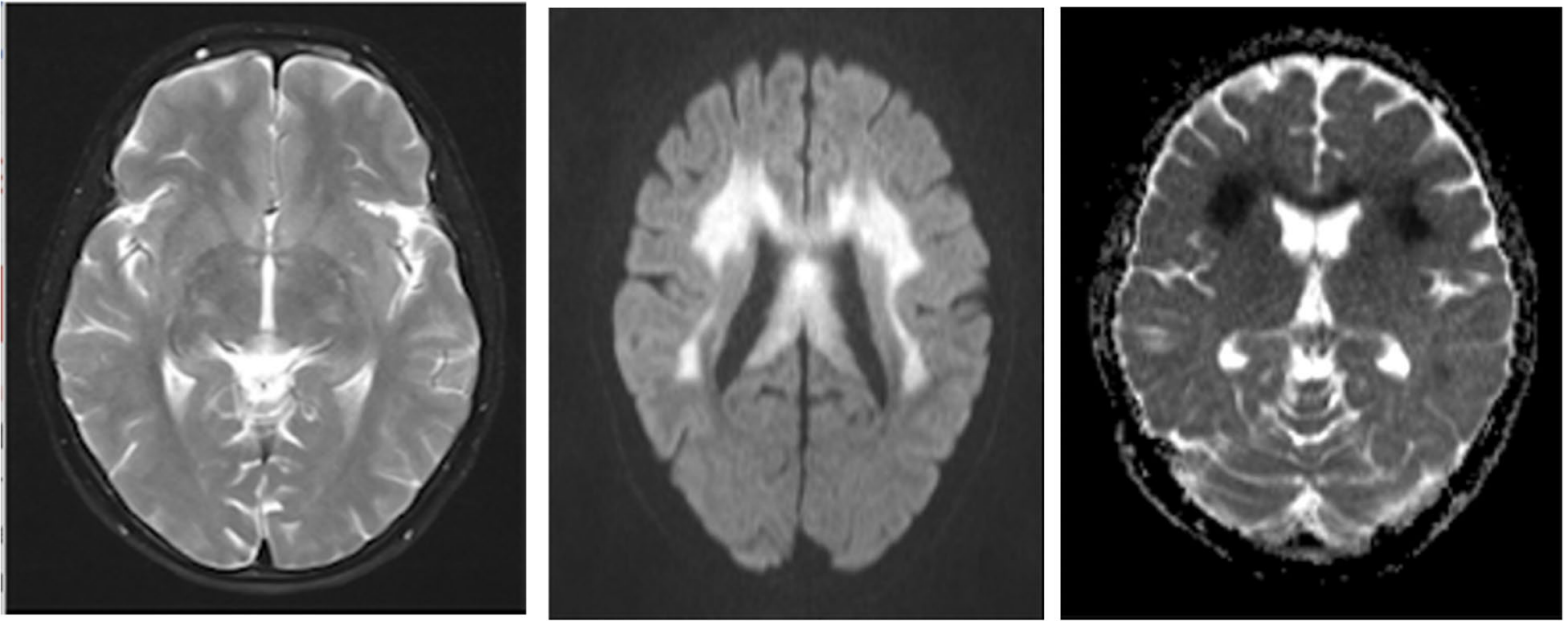
T2W FLAIR MRI sequence showing hyperintensities and corresponding diffusion-weighted imaging showing diffusion restriction involving bilateral central semi-ovale, corona radiata, posterior limb of internal capsule and also genu, body, and splenium of corpus callosum

A 22-year-old lady with poorly differentiated adenocarcinoma of the rectum (cT4bN2) was planned for total neoadjuvant therapy with 6 cycles of modified FOLFOX-6 (oxaliplatin 85 mg/m^2^, leucovorin 400 mg/m^2^, and 5-fluorouracil (5-FU) 400 mg/m^2^ bolus on day 1, followed by 5-FU 2400 mg/m^2^ by continuous infusion over the next 46 h) and long-course chemoradiation therapy (LCCRT) prior to surgery. During the first cycle of mFOLFOX-6, on day 2, she had an episode of transient asymptomatic bradycardia while on the 5-FU infusion. On the third day, while on the 5-FU infusion, she manifested features of encephalopathy including irritability, restlessness, altered sensorium, hemodynamic instability (tachycardia and desaturation), and generalised muscle spasms with associated writhing movement of the hands. She was agitated with tonic posturing of all four limbs, her GCS was E2V4M6 (12/15), and deep tendon reflexes were brisk with mild intention tremor of the right hand. Baseline complete blood count, renal, liver function tests, electrolytes, serum lactates, vitamin B12, and TSH (thyroid-stimulating hormone) levels were within normal limits. Arterial blood gas analysis (ABG) showed features of respiratory alkalosis. Serum ammonia was monitored daily; it rose from 36.4 to 91 mcg% (normal range: males, 27–102 mg/dl; females, 18.7–87 mg/ml) and normalised over a duration of 1 week (Fig. 2). MRI brain with contrast showed T2 FLAIR hyperintensities and diffusion restriction involving centrum semi-ovale bilaterally, corona radiata, the posterior limb of the internal capsule, genu, body, and splenium (Fig. 3). Electroencephalogram (EEG) showed a generalized slowing pattern consistent with metabolic encephalopathy. DPD/DPYD mutation analysis on peripheral blood revealed the presence of a heterozygous mutation in exons 2, 13, and 18. She required a 24-h ICU (intensive care unit) admission for monitoring, where she was administered intravenous thiamine along with oral folic acid, following which she gradually improved and neurological status normalised within a week. She completed LCCRT (with concurrent capecitabine) followed by four cycles of CAPOX chemotherapy in the post-LCCRT waiting period of 12 weeks and then underwent low anterior resection (LAR) with histopathology showing a residual adenocarcinoma ypT3N0. She is disease-free at 25 months of follow-up.

### Case 2

**Fig. 4:**
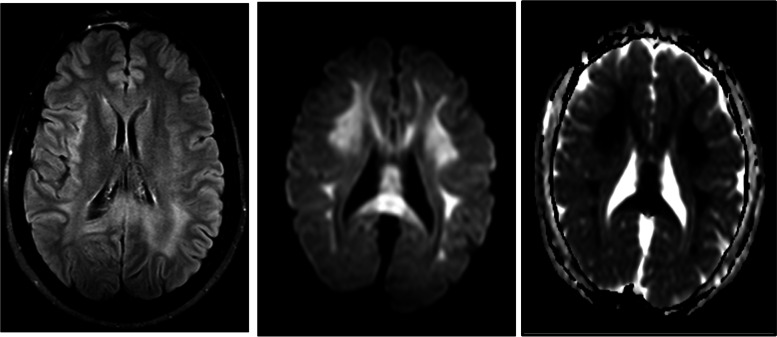
TR hyperintensities with diffusion restriction involving bilateral centrum semi-ovale, corona radiata, deep white matter of bilateral, frontal, and parietal lobes, the corpus callosum, and the posterior limb of the internal capsule

A 30-year-old male with carcinoma rectum cT3N2M0 was treated with LCCRT 50.4 Gy in 28 fractions with concurrent capecitabine (825 mg/m^2^). He was planned for biweekly 5-FU/leucovorin infusions during the 8-week waiting period prior to surgery. He presented with headache, slurred speech, and difficulty in swallowing, on day 4 of the first cycle of 5-FU. His higher mental functions were normal, with a GCS score of E3V4M6 (13/15). He had dysarthria with absent gag reflex and inability to protrude the tongue. His serum ammonia levels were elevated (116 ug% on day 1 and 56 ug% on day 3) (Fig. 2). MRI brain with contrast revealed confluent, symmetrical long TR hyperintensity with diffusion restriction involving bilateral centrum semi-ovale, corona radiata, deep white matter of bilateral frontal and parietal lobes (with involvement of the periventricular deep white matter), the corpus callosum, and the posterior limb of the internal capsule, sparing the subcortical white matter typical of acute toxic leukoencephalopathy (Fig. 4). EEG was normal. DPD/DPYD mutation analysis showed no mutation. He was started on nasogastric feeds and intravenous thiamine. His neurological status improved and normalised with these measures within 48 h of admission. He underwent low anterior resection after 8 weeks; histopathology showed a complete pathological response (ypT0N0). He completed 4 cycles of adjuvant chemotherapy with CAPOX and is disease-free at a follow-up of 23 months.

### Case 3

**Fig. 5:**
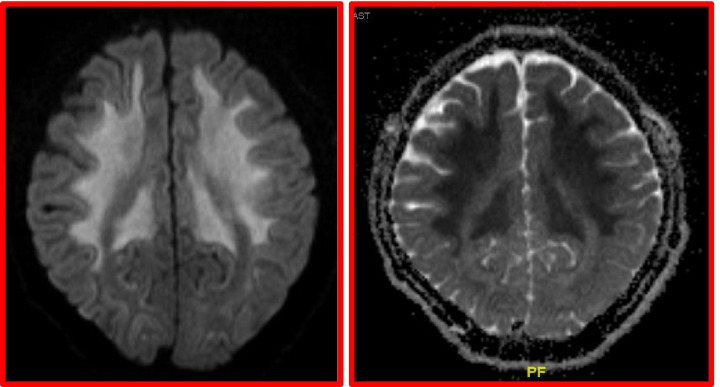
Symmetrical long TR hyperintensity with diffusion restriction of the bilateral frontoparietal deep white matter, the corpus callosum, corona radiata, centrum semi-ovale, and bilateral posterior limbs of the internal capsule

A 34-year-old male with adenocarcinoma of the rectum (staged as cT3N2M0), was planned for LCCRT (50.4 Gy in 28 fractions) with concurrent biweekly infusional 5FU followed by surgery. On day 3 of the third biweekly dose of infusional 5-FU and leucovorin, he presented with giddiness and fatigue. At admission, he was dehydrated and had transient hypotension which was responsive to hydration. Baseline blood investigations were normal except for elevated serum creatinine (1.8 mg/dl). A day later, he then developed neurological symptoms: slurred speech and loss of sensation over lips and mouth with focal seizures (jerky movements involving lower jaw). He was managed conservatively with antiepileptics and intravenous thiamine. Serum ammonia level was 84 ug% on day 1 and 59 ug% after 48 h. MRI brain showed symmetrical long TR hyperintensity with diffusion restriction of bilateral frontoparietal deep white matter, corpus callosum, corona radiata, centrum semi-ovale, and bilateral posterior limbs of internal capsule suggestive of features of drug-induced leukoencephalopathy (Fig. 5), and EEG was normal. DPD/DPYD mutation analysis showed no mutation. He was initiated on intravenous methylprednisolone (6), and his neurological status returned to normal within 3 days with supportive measures. He completed local radiotherapy with no further concurrent chemotherapy.

### Case 4

A 45-year-old lady diagnosed with metastatic poorly differentiated adenocarcinoma of the stomach was planned for palliative chemotherapy with FLOT regimen. During the first cycle, on day 2, while on infusional 5-FU and leucovorin, she developed aphasia and extrapyramidal symptoms. Serum ammonia level was 200 ug% on day 1 and 108 ug% after 1 week (Fig. 2). MRI brain with contrast showed diffuse bilaterally symmetric diffusion restriction involving the deep white matter in the cerebral hemisphere, also involving the corpus callosum and pyramidal tracts, sparing the subcortical U fibres and periventricular white matter, suggestive of drug-induced leukoencephalopathy. She underwent a cerebrospinal fluid (CSF) analysis, inclusive of CSF cytospin, bacterial, AFB, and fungal cultures and multiple PCR for viruses which were all normal. She was initiated on supportive measures and intravenous methylprednisolone for 3 days, following which her neurological status was normalised. No DPD testing was done. She completed 4 cycles of CAPOX chemotherapy with a partial response and is on maintenance capecitabine at 6 months of follow-up.

### Case 5

A 60-year-old gentleman with metastatic adenocarcinoma of the rectum with pulmonary metastases was planned for palliative chemotherapy with FOLFIRINOX (infusional 5-FU, 2400 mg/m^2^; oxaliplatin, 85mg/m^2^; irinotecan, 150 mg/m^2^). Immediately following completion of his second cycle of infusional 5-FU, on day 3, he presented with altered sensorium, agitation, and disorientation. Serum ammonia level was within normal limits on day 1 (56 ug%). MRI brain with contrast showed subtle symmetric long TR hyperintensity in the thalami without any diffusion restriction. He was started on aggressive hydration and intravenous methylprednisolone for 3 days, and his neurological status was normalised within 24 h. He is continuing palliative chemotherapy with CAPOX regimen with bevacizumab.

## Discussion

Our series of five patients illustrates the clinical presentation, radiological features, and treatment of 5-FU-induced acute toxic leukoencephalopathy. This is a rare complication reported in less than 5% of patients receiving 5-FU-based chemotherapy [10]. Also known as *toxic spongiform leukoencephalopathy*, it happens due to progressive structural damage of white matter tracts involved in higher mental function [2]. Other chemotherapeutic agents implicated in toxic leukoencephalopathy include methotrexate, vincristine, ifosfamide, fludarabine, cytarabine, cisplatin, and interferons [11]. The commonest presenting symptoms for toxic leukoencephalopathy are confusion, agitation, ataxia, seizures, or even coma.

The exact mechanism of 5-FU-related neurotoxicity is poorly understood. Koenig et al. attribute the accumulation of fluoroacetate, a product of 5-FU catabolism, leading to the inhibition of the thymidylate synthetase in Krebs’s cycle with impairment of urea cycle, leading to accumulation of ammonia in the blood, which in turn is responsible for the encephalopathy [12].. This accumulation of ammonia in the blood is aggravated by factors like concomitant infection, dehydration, and renal dysfunction [13]. An alternative theory attributed the neurologic adverse effects of 5-FU is the deficiency of thiamine [14]. Exposure to 5-FU increases thiamine pyrophosphate (TPP) level, the active form of thiamine [14, 15]. It leads to an increase in the cellular thiamine metabolism, which in turn exacerbates thiamine deficiency [14]. The similarities in the clinical presentation between 5-FU leukoencephalopathy and Wernicke-Korsakoff syndrome, including ataxia, nystagmus, mental confusion, and cognitive changes, are supportive of this theory.

All our five patients had clinical and radiological findings suggestive of 5-FU-induced encephalopathy (Table 1). This was supported by the presence of hyperammonaemia in all patients (Fig. 2). The median time to onset of symptoms from initiation of 5-FU was 3 days (range: 2–4 days), consistent with earlier reports [13, 16]. The criteria to diagnose 5-FU-related encephalopathy include the following features [7, 9, 17]:

- Development of encephalopathic features during (or) after completion of 5-FU therapy
- Exclusion of other metabolic or physical features causing altered conscious level
- Exclusion of other drugs or concomitant medications

Contrast-enhanced MRI brain with diffusion-weighted imaging (DWI) is the gold standard for radiological diagnosis of toxic leukoencephalopathy [2, 18, 19]. MRI features aiding the diagnosis are diffuse bilateral symmetrical hyperintensities in the T2W flair images with corresponding areas of diffusion restriction in periventricular, deep white matter and corpus callosum with sparing of cortex and subcortical white matter as well as basal ganglia [20]. Two of our patients had focal neurological deficits mimicking a stroke; this entity has been described earlier in a case report, where the patient was treated with thrombolysis [21]. Ideally, reversibility of these findings on MRI can be demonstrated after 4–8 weeks. However, none of our patients underwent a repeat MRI imaging.

Dihydropyrimidine dehydrogenase (DPD) is an enzyme that mediates breakdown of 80% of the administered 5-FU and is distributed in the liver, gastrointestinal mucosa, and peripheral lymphocytes (Fig. 1). Thus, a complete deficiency of this enzyme is known to cause life-threatening or fatal toxicity when a patient is treated with fluoropyrimidine-based chemotherapy [4]. The incidence of DPD deficiency overall among cancer patients has been estimated to be 2.7% and 27% among the largest prior cohort of 30 patients with fluoropyrimidine-related encephalopathy [16, 22]. In our cohort, 3 patients underwent DPYD testing, and one (33%) showed a pathogenic heterozygous mutation.

The dosing of 5-FU also plays a contributory role towards predisposition to 5-FU-induced leukoencephalopathy, with patients receiving doses higher than 1800–2600 mg/m^2^/day, being more susceptible for the same [13]. Kim et al. demonstrated elevated levels of DPD enzyme, among a cohort of patients following treatment with an “intermediate-dose” 5FU and suggested that the transient stagnation of catabolites of 5-FU would play an important role in the development of neurotoxicity [15]. In our cohort, one patient on FLOT chemotherapy for gastric cancer received this “high-dose” 5-FU (2600 mg/m^2^/day), while the rest (*n* = 4) received a lower dose of infusional 5-FU while on the m-FOLFOX 6 (1200 mg/m^2^/day).

There is no current definitive treatment for acute toxic leukoencephalopathy. Prompt identification and withdrawal of the offending agent are the most crucial step, followed by supportive measures like plasma exchange and thiamine infusion (20). Although there is no role for prophylactic steroids, some reports support the use of steroids in the setting of severe encephalopathy and florid MRI changes (20). Three of our patients were given methylprednisolone and responded to the same. All our patients had complete neurological recovery within a week. Though early initiation (within 96 h) of uridine triacetate following life-threatening fluoropyrimidine toxicity has been approved, the access to this drug is difficult [23, 24]. None of our patients underwent a 5-FU rechallenge. Though this is described as an option, the risk of relapse of 5FU encephalopathy is as high as 57%, even if the rechallenge is done in a monitored setting, with lower doses of 5-FU, DPYD testing, and stringent monitoring [16]. We treated four patients with another fluoropyrimidine (capecitabine), and they all successfully completed subsequent treatment without any major toxicity. Our study is limited by its small numbers, lack of information of DPYD status among two patients, and lack of uniformity among treatment approaches and its retrospective nature, which does not account for an accurate assessment of neurological status and hence may lead to underestimation of mild neurological toxicity. Nevertheless, considering the rarity, severity of this entity, and the differences in the pharmacogenomics between Caucasians and Asians, this first series from the Indian subcontinent will increase awareness of fluoropyrimidine-related neurotoxicity and aid early diagnosis and treatment, thus reducing morbidity and mortality.

## Conclusion

5-FU-induced toxic leukoencephalopathy is a rare entity encountered during 5-FU-based chemotherapy for GI malignancies. This should be considered among patients on 5-FU presenting with new onset of neurological symptoms. The typical clinical presentation is encephalopathy, with hyperammonaemia. MRI brain shows characteristic features of diffusion-restricted lesions in the deep cerebral white matter and corpus callosum. Management is conservative and involves immediate discontinuation of the offending drug with supportive measures. Awareness of this clinical-radiological syndrome among oncologists helps early recognition of this entity and timely initiation of appropriate interventions.

## Data Availability

Available on request.

## Abbreviations

ABG: Arterial blood gas analysis
CSF: Cerebrospinal fluid
DPD: Dihydropyrimidine dehydrogenase
DWI: Diffusion-weighted imaging
EC: Ethics committee
EEG: Electroencephalogram
5FU: 5-Fluorouracil
Fd-UMP: Fluorodeoxyuridine monophosphate
FLAIR: Fluid-attenuated inversion recovery
GCS: Glasgow coma scale
GI: Gastrointestinal
Gy: Gray
IRB: Institutional Review Board
ICU: Intensive care unit
LAR: Low anterior resection
LCCRT: Long-course chemoradiation therapy
LV: Leucovorin
MRI: Magnetic resonance imaging
TSH: Thyroid-stimulating hormone
TR: Repetition time

## References

[1] Grem JL. 5-fluorouracil: forty-plus and still ticking. A review of its preclinical and clinical development. Investig New Drugs. 2000;18:299–313. 10.1023/a:1006416410198. 11081567 10.1023/a:1006416410198

[2] Maheen Anwar SS, Mubarak F, Sajjad Z, Azeemuddin M. 5-FU induced acute toxic leukoencephalopathy: early recognition and reversibility on DWI-MRI. J Coll Physicians Surg Pak. 2014;24(Suppl 1):S8–10. 24718016

[3] Layoun ME, Wickramasinghe CD, Peralta MV, Yang EH. Fluoropyrimidine-induced cardiotoxicity: manifestations, mechanisms, and management. Curr Oncol Rep. 2016;18:35. 10.1007/s11912-016-0521-1. 27113369 10.1007/s11912-016-0521-1

[4] Milano G, Etienne MC, Pierrefite V, Barberi-Heyob M, Deporte-Fety R, Renée N. Dihydropyrimidine dehydrogenase deficiency and fluorouracil-related toxicity. Br J Cancer. 1999;79:627–30. 10.1038/sj.bjc.6690098. 10027340 10.1038/sj.bjc.6690098 PMC2362417

[5] Fidai SS, Sharma AE, Johnson DN, Segal JP, Lastra RR. Dihydropyrimidine dehydrogenase deficiency as a cause of fatal 5-fluorouracil toxicity. Autops Case Rep. 2018;8:e2018049. 10.4322/acr.2018.049. 30775324 10.4322/acr.2018.049 PMC6360833

[6] Pirzada NA, Ali II, Dafer RM. Fluorouracil-induced neurotoxicity. Ann Pharmacother. 2000;34:35–8. 10.1345/aph.18425. 10669184 10.1345/aph.18425

[7] Thomas SA, Tomeh N, Theard S. Fluorouracil-induced hyperammonemia in a patient with colorectal cancer. Anticancer Res. 2015;35:6761–3. 26637893

[8] van Kuilenburg ABP. Dihydropyrimidine dehydrogenase and the efficacy and toxicity of 5-fluorouracil. Eur J Cancer. 2004;40:939–50. 10.1016/j.ejca.2003.12.004. 15093568 10.1016/j.ejca.2003.12.004

[9] Naranjo CA, Busto U, Sellers EM, Sandor P, Ruiz I, Roberts EA, et al. A method for estimating the probability of adverse drug reactions. Clin Pharmacol Ther. 1981;30:239–45. 10.1038/clpt.1981.154. 7249508 10.1038/clpt.1981.154

[10] Magge RS, DeAngelis LM. The double-edged sword: neurotoxicity of chemotherapy. Blood Rev. 2015;29:93–100. 10.1016/j.blre.2014.09.012. 25445718 10.1016/j.blre.2014.09.012 PMC5944623

[11] Verstappen CCP, Heimans JJ, Hoekman K, Postma TJ. Neurotoxic complications of chemotherapy in patients with cancer: clinical signs and optimal management. Drugs. 2003;63:1549–63. 10.2165/00003495-200363150-00003. 12887262 10.2165/00003495-200363150-00003

[12] Koenig H, Patel A. Biochemical basis for fluorouracil neurotoxicity. The role of Krebs cycle inhibition by fluoroacetate. Arch Neurol. 1970;23:155–60. 10.1001/archneur.1970.00480260061008. 5430334 10.1001/archneur.1970.00480260061008

[13] Mitani S, Kadowaki S, Komori A, Sugiyama K, Narita Y, Taniguchi H, et al. Acute hyperammonemic encephalopathy after fluoropyrimidine-based chemotherapy: a case series and review of the literature. Medicine. 2017;96:e6874. 10.1097/MD.0000000000006874. 28562536 10.1097/MD.0000000000006874 PMC5459701

[14] Heier MS, Dornish JM. Effect of the fluoropyrimidines 5-fluorouracil and doxifluridine on cellular uptake of thiamin. Anticancer Res. 1989;9:1073–7. 2530931

[15] Kim Y-A, Chung HC, Choi HJ, Rha SY, Seong JS, Jeung H-C. Intermediate dose 5-fluorouracil-induced encephalopathy. Jpn J Clin Oncol. 2006;36:55–9. 10.1093/jjco/hyi214. 16436463 10.1093/jjco/hyi214

[16] Boilève A, Thomas L, Lillo-Le Louët A, Gaboriau L, Chouchana L, Ducreux M, et al. 5-fluorouracil-induced hyperammonaemic encephalopathy: a French national survey. Eur J Cancer. 2020;129:32–40. 10.1016/j.ejca.2020.01.019. 32120273 10.1016/j.ejca.2020.01.019

[17] Cordier P-Y, Nau A, Ciccolini J, Oliver M, Mercier C, Lacarelle B, et al. 5-FU-induced neurotoxicity in cancer patients with profound DPD deficiency syndrome: a report of two cases. Cancer Chemother Pharmacol. 2011;68:823–6. 10.1007/s00280-011-1666-0. 21553285 10.1007/s00280-011-1666-0

[18] Acharya G, Cruz Carreras MT, Rice TW. 5-FU-induced leukoencephalopathy with reversible lesion of splenium of corpus callosum in a patient with colorectal cancer. BMJ Case Rep. 2017;2017. 10.1136/bcr-2017-222030. 10.1136/bcr-2017-222030 PMC5720253 29167217

[19] Akitake R, Miyamoto S, Nakamura F, Horimatsu T, Ezoe Y, Muto M, et al. Early detection of 5-FU-induced acute leukoencephalopathy on diffusion-weighted MRI. Jpn J Clin Oncol. 2011;41:121–4. 10.1093/jjco/hyq157. 20926409 10.1093/jjco/hyq157

[20] Lee W-W, Kim J-S, Son KR, Kwon H-M. Atypical diffusion-restricted lesion in 5-fluorouracil encephalopathy. AJNR Am J Neuroradiol. 2012;33:E102–3. 10.3174/ajnr.A2781. 22403780 10.3174/ajnr.A2781 PMC7965488

[21] Kinno R, Kii Y, Uchiyama M, Owan Y, Yamazaki T, Fukui T. 5-fluorouracil–induced leukoencephalopathy with acute stroke-like presentation fulfilling criteria for recombinant tissue plasminogen activator therapy. J Stroke Cerebrovasc Dis. 2014;23:387–9. 10.1016/j.jstrokecerebrovasdis.2013.01.014. 23422344 10.1016/j.jstrokecerebrovasdis.2013.01.014

[22] Etienne MC, Lagrange JL, Dassonville O, Fleming R, Thyss A, Renée N, et al. Population study of dihydropyrimidine dehydrogenase in cancer patients. J Clin Oncol. 1994;12:2248–53. 10.1200/JCO.1994.12.11.2248. 7964939 10.1200/JCO.1994.12.11.2248

[23] Lampropoulou DI, Laschos K, Amylidi A-L, Angelaki A, Soupos N, Boumpoucheropoulos S, et al. Fluoropyrimidine-induced toxicity and DPD deficiency.. A case report of early onset, lethal capecitabine-induced toxicity and mini review of the literature. Uridine triacetate: efficacy and safety as an antidote. Is it accessible outside USA? J Oncol Pharm Pract. 2020;26:747–53. 10.1177/1078155219865597. 31382864 10.1177/1078155219865597

[24] Ison G, Beaver JA, McGuinn WD, Palmby TR, Dinin J, Charlab R, et al. FDA approval: uridine triacetate for the treatment of patients following fluorouracil or capecitabine overdose or exhibiting early-onset severe toxicities following administration of these drugs. Clin Cancer Res. 2016;22:4545–9. 10.1158/1078-0432.CCR-16-0638. 27401247 10.1158/1078-0432.CCR-16-0638

